# S-1 and oxaliplatin versus tegafur-uracil and leucovorin as post-operative adjuvant chemotherapy in patients with high-risk stage III colon cancer: updated 5-year survival of the phase III ACTS-CC 02 trial

**DOI:** 10.1016/j.esmoop.2021.100077

**Published:** 2021-03-11

**Authors:** J. Watanabe, S. Sasaki, T. Kusumoto, Y. Sakamoto, K. Yoshida, N. Tomita, A. Maeda, J. Teshima, M. Yokota, C. Tanaka, J. Yamauchi, H. Uetake, M. Itabashi, K. Takahashi, H. Baba, K. Kotake, N. Boku, K. Aiba, S. Morita, N. Takenaka, K. Sugihara

**Affiliations:** 1 Gastroenterological Center, Yokohama City University Medical Center, Kanagawa, Japan; 2 Department of Surgical Oncology, Japanese Red Cross Medical Center, Tokyo, Japan; 3 Department of Gastrointestinal Surgery and Clinical Research Institute Cancer Research Division, National Kyushu Medical Center, Fukuoka, Japan; 4 Department of Gastroenterological Surgery, Hirosaki University Graduate School of Medicine, Aomori, Japan; 5 Department of Surgical Oncology, Gifu University Graduate School of Medicine, Gifu, Japan; 6 Division of Lower GI Surgery, Department of Surgery, Hyogo College of Medicine, Hyogo, Japan; 7 Department of Surgery, Ogaki Municipal Hospital, Gifu, Japan; 8 Department of Gastrointestinal Surgery, Iwate Prefectural Central Hospital, Iwate, Japan; 9 Department of General Surgery, Kurashiki Central Hospital, Okayama, Japan; 10 Department of Surgery, Gifu Prefectural General Medical Center, Gifu, Japan; 11 Department of Surgery, Sendai Kousei Hospital, Miyagi, Japan; 12 Department of Specialized Surgeries, Tokyo Medical and Dental University, Tokyo, Japan; 13 Department of Surgery, Institute of Gastroenterology, Tokyo Women’s Medical University, Tokyo, Japan; 14 Department of Surgery, Tokyo Metropolitan Cancer and Infectious Diseases Center Komagome Hospital, Tokyo, Japan; 15 Department of Gastroenterological Surgery, Graduate School of Medical Sciences, Kumamoto University, Kumamoto, Japan; 16 Department of Surgery, Sano City Hospital, Tochigi, Japan; 17 Department of Gastrointestinal Medical Oncology, National Cancer Center Hospital, Tokyo, Japan; 18 Division of Clinical Oncology/Hematology, Department of Internal Medicine, The Tokyo Jikei University School of Medicine, Tokyo, Japan; 19 Department of Biomedical Statistics and Bioinformatics, Kyoto University Graduate School of Medicine, Kyoto, Japan; 20 Clinical Research & Pharmacoepidemiology Department, Medical Affairs Division, Taiho Pharmaceutical Co., Ltd, Tokyo, Japan; 21 Tokyo Medical and Dental University, Tokyo, Japan

**Keywords:** SOX, UFT/LV, colorectal cancer, L-OHP, risk group

## Abstract

**Background:**

The ACTS-CC 02 trial demonstrated that S-1 plus oxaliplatin (SOX) was not superior to tegafur-uracil and leucovorin (UFT/LV) in terms of disease-free survival (DFS) as adjuvant chemotherapy for high-risk stage III colon cancer (any T, N2, or positive nodes around the origin of the feeding arteries). We now report the final overall survival (OS) and subgroup analysis according to the pathological stage (TNM 7th edition) for treatment efficacy.

**Patients and methods:**

Patients who underwent curative resection for pathologically confirmed high-risk stage III colon cancer were randomly assigned to receive either UFT/LV (300 mg/m^2^ of UFT and 75 mg/day of LV on days 1-28, every 35 days, five cycles) or SOX (100 mg/m^2^ of oxaliplatin on day 1 and 80 mg/m^2^/day of S-1 on days 1-14, every 21 days, eight cycles). The primary endpoint was DFS and the patients’ data were updated in February 2020.

**Results:**

A total of 478 patients in the UFT/LV group and 477 patients in the SOX group were included in the final analysis. With a median follow-up time of 74.3 months, the 5-year DFS rate was 55.2% in the UFT/LV group and 58.1% in the SOX group [stratified hazard ratio (HR) 0.92; 95% confidence interval (CI) 0.76-1.11; *P* = 0.3973], and the 5-year OS rates were 78.3% and 79.1%, respectively (stratified HR 0.97; 95% CI 0.76-1.24; *P* = 0.8175). In the subgroup analysis, the 5-year OS rates in patients with T4N2b disease were 51.0% and 64.1% in the UFT/LV and SOX groups, respectively (HR 0.72; 95% CI 0.40-1.31).

**Conclusion:**

Our final analysis reconfirmed that SOX as adjuvant chemotherapy is not superior to UFT/LV in terms of DFS in patients with high-risk stage III colon cancer. The 5-year OS rate was similar in the UFT/LV and SOX groups.

## Highlights

• This final analysis reconfirmed that SOX is not superior to UFT/LV in high-risk stage III colon cancer in terms of DFS.
• With a median follow-up time of 74.3 months, the 5-year OS rate was similar in both groups.
• The 5-year OS rates in patients with T4N2b disease were 51.0% and 64.1% in the UFT/LV and SOX groups, respectively.

## Introduction

Fluoropyrimidine plus oxaliplatin therapy is widely used as standard postoperative adjuvant chemotherapy for patients with stage III colon cancer worldwide. Among previous three phase III studies examining the superiority of oxaliplatin-based therapy, the NO16968 trial compared intravenous infusion of 5-fluorouracil (5-FU)/leucovorin (LV) with oral capecitabine and oxaliplatin (CAPOX), and the MOSAIC and NSABP C-07 trials compared 5-FU/LV with a combination therapy, including 5-FU/LV and oxaliplatin.^1^, ^2^, ^3^ In Japan, oral fluoropyrimidines, such as capecitabine, tegafur-uracil and LV (UFT/LV), and S-1, are preferred because they have demonstrated favorable outcomes in patients with stage III colon cancer in Japanese randomized trials, and their treatment is convenient and associated with only mild toxicity.^4^, ^5^, ^6^, ^7^ However, stage III includes patient subgroups showing very poor outcomes, such as the N2 disease.^4^^,^^7^ According to the Japanese Classification of Colorectal Carcinoma (7th edition), the main lymph nodes are defined as lymph nodes around the origin of the feeding arteries (i.e. the ileocolic, right colic, middle colic, or inferior mesenteric artery; Supplementary Figure S1, available at https://doi.org/10.1016/j.esmoop.2021.100077). N1 tumors with metastasis to the main lymph nodes have been associated with poorer outcomes than N2a tumors without metastasis to the main lymph nodes.^8^ Therefore, stage III disease with N2 and N1 including metastasis to the main lymph nodes is classified as a high-risk group. There remains room for improvement in the outcomes of patients with such high-risk stage III disease, and more aggressive regimens including oxaliplatin are expected to be effective in Japan as well.

The ACTS-CC trial showed that S-1 was noninferior to UFT/LV as adjuvant chemotherapy for stage III colon cancer.^4^ As first-line treatment for metastatic colorectal cancer, S-1 plus oxaliplatin (SOX) was reported to be noninferior to CAPOX.^9^ However, the efficacy of SOX as adjuvant chemotherapy for colon cancer has not yet been established. We therefore conducted the ACTS-CC 02 trial to validate the therapeutic efficacy of oral fluoropyrimidine plus oxaliplatin adjuvant chemotherapy in patients with high-risk stage III (‘any T, N2’ or ‘any T, positive main lymph nodes’) colon cancer in Japan. We also sought to verify the efficacy of adjuvant chemotherapy with SOX in patients with colon cancer.

During the primary analysis with a median follow-up period of 58.4 months, SOX was not shown to be superior to UFT/LV in terms of disease-free survival (DFS) for primary endpoint in patients with high-risk stage III colon cancer [hazard ratio (HR) 0.90, 95% confidence interval (CI) 0.74-1.09], when there were only 187 events (19.6%) among 955 patients in terms of overall survival (OS).^10^ We now report the results of the updated and comprehensive analyses of OS, which were based on preliminary data during the primary analysis, and exploratory analyses according to the pathological stage (TNM 7th edition).

## Methods

### Study design and patients

The ACTS-CC 02 trial is an open-label, multicenter, randomized phase III trial conducted in accordance with the ethical principles of the Declaration of Helsinki and was in compliance with the Japanese ethical guidelines for clinical studies. The study was approved by the Institutional Review Board of each participating institution. All patients provided written informed consent before enrollment. This trial is registered with the Japan Pharmaceutical Information Center (JapicCTI-101073) and Japan Registry of Clinical Trials (jRCTs031180351).

Patients aged 20 to 80 years with curatively resected high-risk stage III (‘any T, N2’ or ‘any T, positive main lymph nodes’) colon cancer were randomly assigned in a 1 : 1 ratio to the UFT/LV or SOX group, using the minimization method with the following stratification factors: tumor location (colon or upper rectum), the number of positive lymph nodes (four to six positive lymph nodes and main lymph nodes metastasis negative, or seven or more positive lymph nodes or main lymph nodes metastasis positive), and institution.

### Procedures

In the UFT/LV group, UFT (300 mg/m^2^) and LV (75 mg/body) were orally administered daily in three divided doses for 28 consecutive days followed by a 7-day rest for a total of five cycles (25 weeks). In the SOX group, S-1 (80 mg/m^2^ daily) was orally administered in two divided doses, after dinner on day 1 to after breakfast on day 15 followed by a 7-day rest. Oxaliplatin (100 mg/m^2^) was infused intravenously on day 1 of each cycle. A total eight cycles (24 weeks) were administered. After completing the study treatment, patients were followed up according to a predefined surveillance schedule. The methods used in this trial have been described in detail previously.^11^

### Outcomes

The primary endpoint was DFS and secondary endpoints were relapse-free survival (RFS), OS, and adverse events. DFS was defined as the period from the date of enrollment to the date of recurrence, secondary cancer, or death from any cause, whichever occurred first. Secondary cancer included metachronous cancers developing in not only the colorectum but also other organs.

### Statistical analysis

The 3-year DFS rate was estimated to be 65% for the UFT/LV group and 71.5% for the SOX group (HR 0.78). Five hundred and ten events in a total of 1186 enrolled patients were required to provide a statistical power of 80% (2-sided alpha value of 0.05) for detecting a difference between the groups after 3 years of follow-up. The primary analysis was conducted using the full analysis set on an intention-to-treat basis. We estimated time-dependent events using the Kaplan–Meier method. The stratified log-rank test using stratification factors excluding institution was used to compare the SOX with the UFT/LV group. All statistical analyses were performed using SAS version 9.13 or higher (SAS Institute, Cary, NC, USA).

## Results

**Figure 1:**
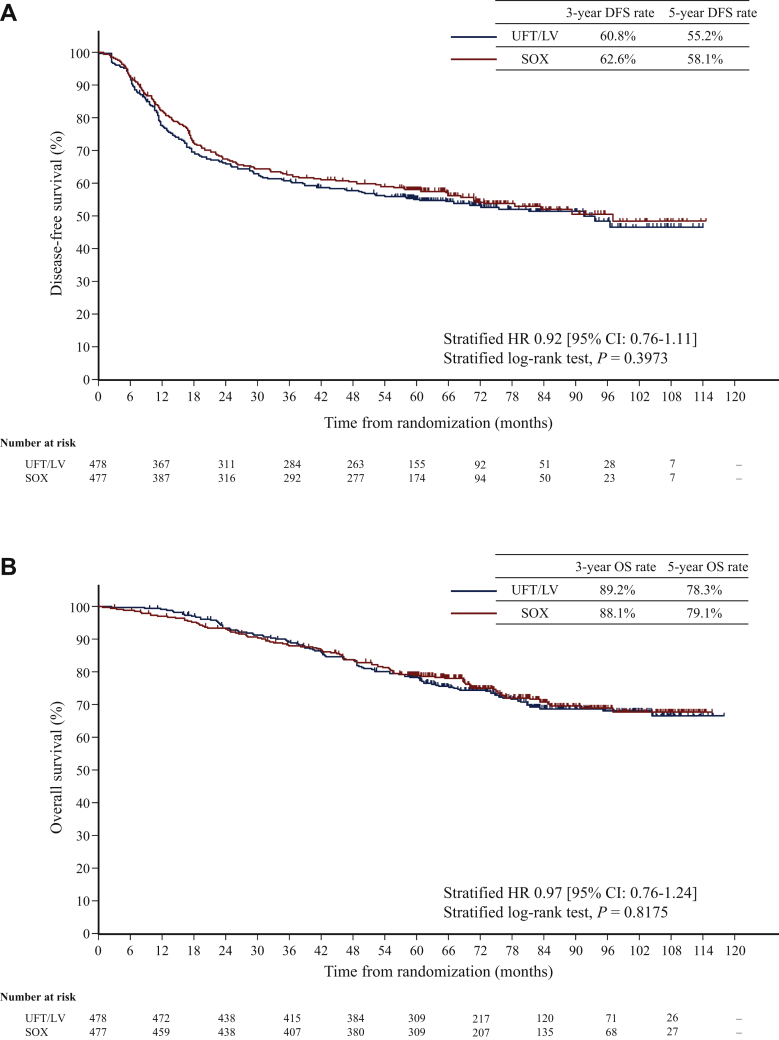
Kaplan–Meier curves for (A) disease-free survival and (B) overall survival. CI, confidence interval; DFS, disease-free survival; HR, hazard ratio; LV, leucovorin; SOX, S-1 plus oxaliplatin; UFT, tegafur-uracil.

From 1 April 2010 through 17 October 2014, a total of 966 patients were enrolled in 260 institutions in Japan. Enrollment was discontinued before the target number was reached owing to slow registration. Patients were randomly assigned to the UFT/LV and SOX groups; patients who withdrew informed consent (nine patients) or violated the Japanese ethical guidelines for clinical studies (two patients) were excluded. Finally, a total of 478 patients in the UFT/LV group and 477 patients in the SOX group were included in the updated analysis (Supplementary Figure S2, available at https://doi.org/10.1016/j.esmoop.2021.100077). Demographic characteristics were well balanced between the groups (Table 1). A total of 865/955 (90.6%) patients underwent D3 lymph node dissection and the others went through a D2 lymph node dissection. Median number of lymph nodes examined was 21 (range 0-107). The mean relative dose intensities (RDIs) were 83.1% for UFT and 84.7% for LV in the UFT/LV group and 74.9% for S-1 and 73.6% for oxaliplatin in the SOX group. Furthermore, the mean RDI was lower in female than in male patients for both groups. The mean RDIs were 80.7% and 85.1% for UFT, 82.9% and 86.4% for LV, 70.5% and 78.4% for S-1, and 69.2% and 77.2% for oxaliplatin in female and male patients, respectively. As of 28 February 2020, the final cut-off date for data collection, the median follow-up period was 74.3 months (range 3.1-117.7). DFS events occurred in 431/955 (45.1%) patients. The 5-year DFS was 55.2% (95% CI 50.6%-59.6%) in the UFT/LV group and 58.1% (95% CI 53.5%-62.4%) in the SOX group. The stratified HR for DFS was 0.92 (95% CI 0.76-1.11; stratified log-rank test, *P* = 0.3973; Figure 1A). The 5-year RFS was 59.0% (95% CI 58.2-66.9) in the UFT/LV group and 61.9% (95% CI 60.5-69.2) in the SOX group. The stratified HR for RFS was 0.92 (95% CI 0.75-1.12; stratified log-rank test, *P* = 0.3956). OS analysis was conducted on the basis of 254 deaths (26.2%) out of 955 patients, representing an increase of 78 deaths compared with the primary analysis. The causes of death were progressive disease in 217 patients, secondary cancer in 11 patients, and other reasons in 26 patients. The 5-year OS rate was 78.3% (95% CI 74.2-81.8) in the UFT/LV group and 79.1% (95% CI 75.1-82.5) in the SOX group. The stratified HR for OS was 0.97 (95% CI 0.76-1.24; stratified log-rank test, *P* = 0.8175; Figure 1B).

**Table 1:** Baseline characteristics

Characteristics	UFT/LV (n = 478)	SOX (n = 477)
	n (%)	n (%)
Age, years		
Median (range)	65.5 (32-80)	65 (26-80)
<70	311 (65.1)	329 (69.0)
≥70	167 (34.9)	148 (31.0)
Sex		
Male	255 (53.3)	263 (55.1)
Female	223 (46.7)	214 (44.9)
ECOG PS		
0	455 (95.2)	443 (92.9)
1	23 (4.8)	34 (7.1)
Tumor location		
Right colon	191 (40.0)	182 (38.2)
Left colon	132 (27.6)	141 (29.6)
Rectum^a^	155 (32.4)	154 (32.2)
Histological type		
Papillary, tubular	425 (88.9)	427 (89.5)
Poorly, mucinous, signet	53 (11.1)	50 (10.5)
Depth of tumor invasion (TNM 7th)		
T1	8 (1.7)	6 (1.3)
T2	17 (3.6)	21 (4.4)
T3	296 (61.9)	294 (61.6)
T4a	126 (26.4)	135 (28.3)
T4b	31 (6.5)	21 (4.4)
Lymphatic invasion		
(−)	52 (10.9)	54 (11.3)
(+)	426 (89.1)	423 (88.7)
Venous invasion		
(−)	98 (20.5)	98 (20.5)
(+)	380 (79.5)	379 (79.5)
Scope of LN dissection		
D2	56 (11.7)	34 (7.1)
D3	422 (88.3)	443 (92.9)
No. of LNs examined		
Median (range)	21 (0-96)	22 (0-107)
<12	62 (13.0)	49 (10.2)
≥12	416 (87.0)	428 (89.7)
No. of LN metastases (stratification factor)		
Median (range)	5 (1-22)	5 (1-39)
4-6 and main LN negative	286 (59.8)	285 (59.7)
≥7 or main LN positive	192 (40.2)	192 (40.3)
LN metastasis (TNM 7th)		
N1a (1 positive LN)	2 (0.4)	4 (0.8)
N1b (2-3 positive LN)	17 (3.6)	21 (4.4)
N2a (4-6 positive LN)	320 (66.9)	318 (66.7)
N2b (≥7 positive LN)	139 (29.1)	134 (28.1)
Stage (TNM 7th)		
IIIA	6 (1.3)	6 (1.3)
IIIB	245 (51.3)	234 (49.1)
IIIC	227 (47.5)	237 (49.7)
Risk group (TNM 7th)		
T3N2a	209 (43.7)	190 (39.8)
T4N2a	92 (19.2)	106 (22.2)
T3N2b	73 (15.3)	87 (18.2)
T4N2b	61 (12.8)	43 (9.0)
Other	43 (9.0)	51 (10.7)

ECOG, Eastern Cooperative Oncology Group; LN, lymph node; LV, leucovorin; PS, performance status; SOX, S-1 plus oxaliplatin; UFT, tegafur-uracil.

a Including patients in whom the lower edge of the tumor is in the upper rectum proximal to the peritoneal reflection.

**Figure 2:**
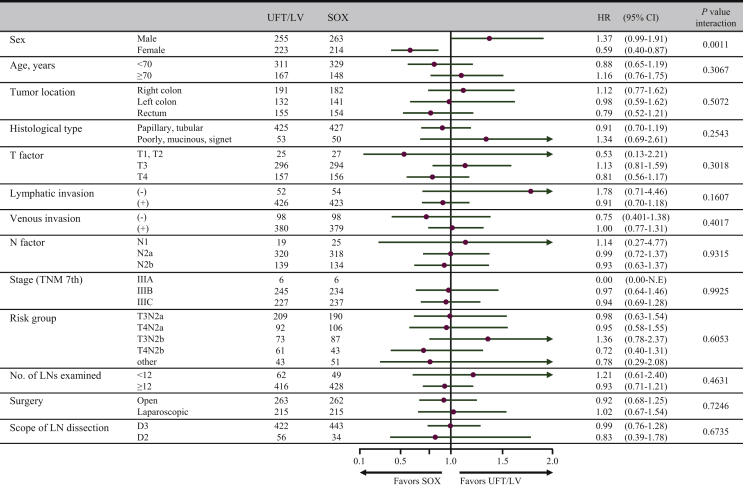
Subgroup analyses of overall survival. CI, confidence interval; HR, hazard ratio; LN, lymph node; LV, leucovorin; SOX, S-1 plus oxaliplatin; UFT, tegafur-uracil.

In the subgroup analysis of DFS, no significant interactions were identified between the major baseline characteristics and the therapeutic effects of UFT/LV and SOX (Supplementary Figure S3, available at https://doi.org/10.1016/j.esmoop.2021.100077). However, a significant interaction was observed between the therapeutic effects and sex in the subgroup analysis of OS (*P* = 0.0011; Figure 2).

**Figure 3:**
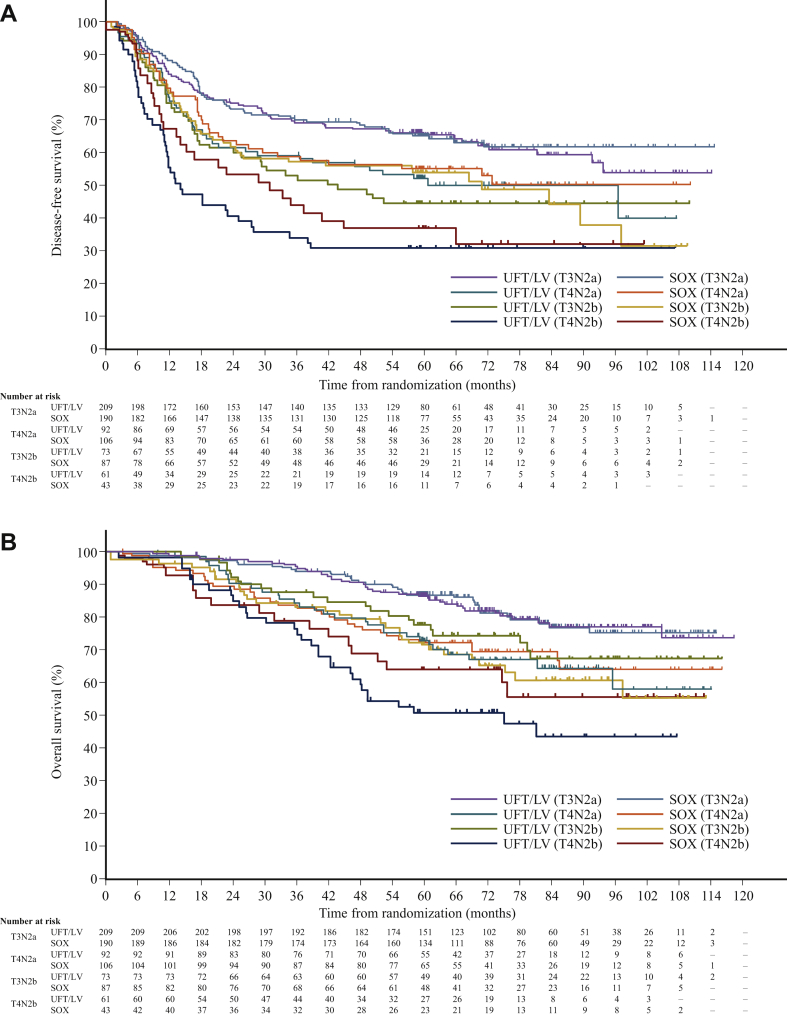
Kaplan–Meier curves according to risk group (TNM 7th) for (A) disease-free survival and (B) overall survival. Annotation: The number of patients with T1 or T2 disease, or those with T disease including N1 metastasis was <20. Therefore, data on these patients are not included in the figures. LV, leucovorin; SOX, S-1 plus oxaliplatin; UFT, tegafur-uracil.

In an exploratory analysis, the 3- and 5-year DFS and OS rates according to the TNM 7th stage, T factor, and N factor subgroups are shown in Table 2. Particularly, DFS and OS depended on the T and N diseases, and the associated curves were clearly separable (Table 2; Figure 3). DFS and OS rates at 5 years in T4N2b patients were 31.1% (95% CI 20.1-42.9) and 51.0% (95% CI 37.6-62.8), respectively, in the UFT/LV group and 37.2% (95% CI 23.1-51.3) and 64.1% (95% CI 47.7-76.6), respectively, in the SOX group (HR 0.72; 95% CI 0.40-1.31; Table 2; Figure 3).

**Table 2:** DFS and OS according to the TNM 7th stage, T factor, and N factor subgroups

Subgroups	Regimen	*N*	Disease-free survival			Overall survival		
			3-year DFS, %	5-year DFS, %	HR (95% CI)	3-year OS, %	5-year OS, %	HR (95% CI)
Stage IIIB	UFT/LV	245	69.4	64.5	1.02 (0.76-1.36)	94.6	86.5	0.97 (0.64-1.46)
	SOX	234	68.6	63.2		93.5	86.8	
Stage IIIC	UFT/LV	227	50.9	44.5	0.86 (0.67-1.10)	83.0	68.8	0.94 (0.69-1.28)
	SOX	237	55.8	51.8		82.4	70.9	
T3	UFT/LV	296	64.5	59.3	0.98 (0.77-1.26)	92.8	84.0	1.13 (0.81-1.59)
	SOX	294	65.3	60.3		91.3	82.6	
T4	UFT/LV	157	50.7	45.4	0.87 (0.65-1.19)	81.2	65.2	0.81 (0.56-1.17)
	SOX	156	54.4	50.5		82.0	70.8	
N2a	UFT/LV	320	67.1	62.0	0.93 (0.73-1.20)	92.0	83.2	0.99 (0.72-1.37)
	SOX	318	66.8	62.9		89.8	82.0	
N2b	UFT/LV	139	46.0	39.4	0.84 (0.61-1.15)	82.6	66.2	0.93 (0.63-1.37)
	SOX	134	54.7	49.2		82.4	70.3	
T3N2a	UFT/LV	209	69.4	65.6	0.95 (0.69-1.32)	95.1	86.6	0.98 (0.63-1.54)
	SOX	190	70.3	65.3		94.1	86.9	
T4N2a	UFT/LV	92	59.4	52.5	0.94 (0.63-1.41)	84.5	73.1	0.95 (0.58-1.55)
	SOX	106	57.4	55.5		83.0	72.4	
T3N2b	UFT/LV	73	53.3	44.9	0.87 (0.56-1.33)	87.7	77.8	1.36 (0.78-2.37)
	SOX	87	59.0	54.1		83.2	71.9	
T4N2b	UFT/LV	61	34.4	31.1	0.81 (0.50-1.31)	74.9	51.0	0.72 (0.40-1.31)
	SOX	43	44.2	37.2		78.9	64.1	

The number of patients with T1 and T2 disease, N1 metastasis, and at stage IIIA was <21 in each group. Therefore, data on these patients are not shown.

CI, confidence interval; DFS, disease-free survival; HR, hazard ratio; LV, leucovorin; OS, overall survival; SOX, S-1 plus oxaliplatin; UFT, tegafur-uracil.

## Discussion

In this study, we updated DFS and OS data from the ACTS-CC 02 trial after long-term follow-up, where the median follow-up period was 74.3 months. This final analysis reconfirmed that in terms of DFS, SOX is not superior to UFT/LV in patients with high-risk stage III colon cancer. The difference in the 5-year DFS rate was only 2.9 percentage points in the SOX group compared with the UFT/LV group (HR 0.92, 95% CI 0.76-1.11). The 5-year OS rates were also similar in the UFT/LV and SOX groups (HR 0.97, 95% CI 0.76-1.24).

As for DFS, the HR was lower in T4 disease than in T3 disease (number of patients enrolled with T1 and T2 disease was too small) and was even lower when with the N factors. As for OS, similar relationships between efficacy of SOX and T/N factors were observed; however, UFT/LV showed better OS than SOX in T3 disease. Although the results of subgroup analysis were not statistically significant due to the small number of patients, SOX had a favorable efficacy, in terms of DFS and OS, in patients with more advanced disease associated with factors such as T4N2b, and this might be a target population for SOX therapy. Further investigation in this population is required to elucidate the real efficacy of SOX in T4N2b population.

The 5-year OS rate of stage IIIB and stage IIIC patients in the UFT/LV group of this study was comparable to that seen in the ACTS-CC trial that compared S-1 and UFT/LV in Japanese stage III colon cancer patients. The 5-year OS rate of stage IIIC patients in the SOX group was slightly higher than that of the patients in the ACTS-CC trial.^12^ In our study, 95.4% of the cases were N2 patients, whereas in ACTS-CC trial only 21.4% of the cases were N2 patients. While patients with stage IIIB and IIIC cancer in the ACTS-CC trial were a group with a relatively good prognosis that included many N1 cases, the 5-year OS rate of the UFT/LV group was comparable. We believe this is related to progress in the treatment of metastatic colorectal cancer in the past decade. Median survival time now exceeds 30 months courtesy of molecular-targeted agents, such as ramucirumab and aflibercept, as well as regorafenib and trifluridine/tipiracil that are typically late-line drugs.^13^, ^14^, ^15^, ^16^ In general, it is difficult to demonstrate efficacy of postsurgical adjuvant chemotherapy on OS, despite a more obvious effect on DFS.^17^ Accordingly, in our study, while the difference in 5-year DFS between the two groups was 2.9 percentage points, the difference in 5-year OS was only 0.8 percentage points.

In addition, circulating tumor DNA (ctDNA) testing for the detection of minimal residual disease targeting metastatic colorectal cancer has been effective for the detection of patient groups with a high recurrence risk. It was shown that the ctDNA-positive group has a significantly higher recurrence rate than the negative group, and the ctDNA positivity rate was high in cases with more advanced disease.^18^ If, by combining the conventional TNM staging with ctDNA gene testing, precise identification of recurrence risk becomes possible, in the future it may be conceivable to select treatment regimens depending on the recurrence risk.

The subgroup analysis of DFS in our study did not show any differences according to sex; however, the subgroup analysis of OS showed that SOX significantly improves the OS of female patients. Conversely, RDI was lower for female than for male patients. In the subgroup analysis of OS in the SOFT study that examined the first-line treatment of metastatic colorectal cancer, despite there being no significant interactions, SOX using oxaliplatin 130 mg/m^2^ was favorable compared with FOLFOX (folinic acid, fluorouracil, and oxaliplatin) in female patients, and the RDI was lower for female patients in both the SOX and FOLFOX groups.^19^^,^^20^ Furthermore, in a study comparing the SOX regimen using 100 mg/m^2^ with S-1 plus cisplatin as a first-line treatment for advanced gastric cancer, the RDI for SOX was lower in female patients, but there was no difference in the RDI of S-1 plus cisplatin according to patient sex, nor was there any difference in the OS on the basis of sex.^21^ These reports suggest that SOX may contribute to the OS in female patients, but the reasons underlying this association remains unclear.

Our current study had some limitations that should be considered while interpreting the findings. First, all patients enrolled in this study were Japanese and 90.6% of patients underwent D3 lymph node dissection; therefore, the results may not be generalizable to patients of other ethnicities. Second, 510 DFS events were required to provide a statistical power of 80% for detecting a difference between the groups. Because the target number of patients was not reached, 79 required events were not enough, ultimately leading to an underpowered study even after a median follow-up of >6 years. Finally, the ACTS-CC 02 trial compared different types of oral fluoropyrimidines, namely, S-1 and UFT/LV. It was not a study that tested the synergistic effect of adding oxaliplatin to fluoropyrimidine. At the time of planning the ACTS-CC 02 trial, the ACTS-CC trial was ongoing to demonstrate the noninferiority of S-1 to UFT/LV.^4^ Therefore, S-1 could not be used as the control arm. However, the NO16968 trial compared intravenous infusion 5-FU/LV and oral CAPOX rather than oral capecitabine and CAPOX and is positioned as a study that tested the effect of adding oxaliplatin.^2^ Because our objective was to develop a combination therapy consisting of oxaliplatin and oral fluoropyrimidine, which is preferred by Japanese high-risk stage III colorectal cancer patients, the difference in the fluoropyrimidines was not a major concern.

## Conclusions

This final analysis reconfirmed that SOX is not superior to UFT/LV in patients with high-risk stage III colon cancer in terms of DFS. The 5-year OS rate was similar in the UFT/LV and SOX groups. The oxaliplatin-based regimen could be more effective for DFS and OS in patients with advanced disease, such as T4N2b.
