# Supplementary material for: S-1 and oxaliplatin versus tegafur-uracil and leucovorin as post-operative adjuvant chemotherapy in patients with high-risk stage III colon cancer: updated 5-year survival of the phase III ACTS-CC 02 trial
Source: ESMO Open. 2021 Mar 11;6(2):100077. doi: 10.1016/j.esmoop.2021.100077 (PMC7966838; doi:10.1016/j.esmoop.2021.100077)

**Supplementary Figure**

**Figure S1: Main lymph nodes defined in the Japanese Classification of Colorectal Carcinoma, 7^th^ edition**

**
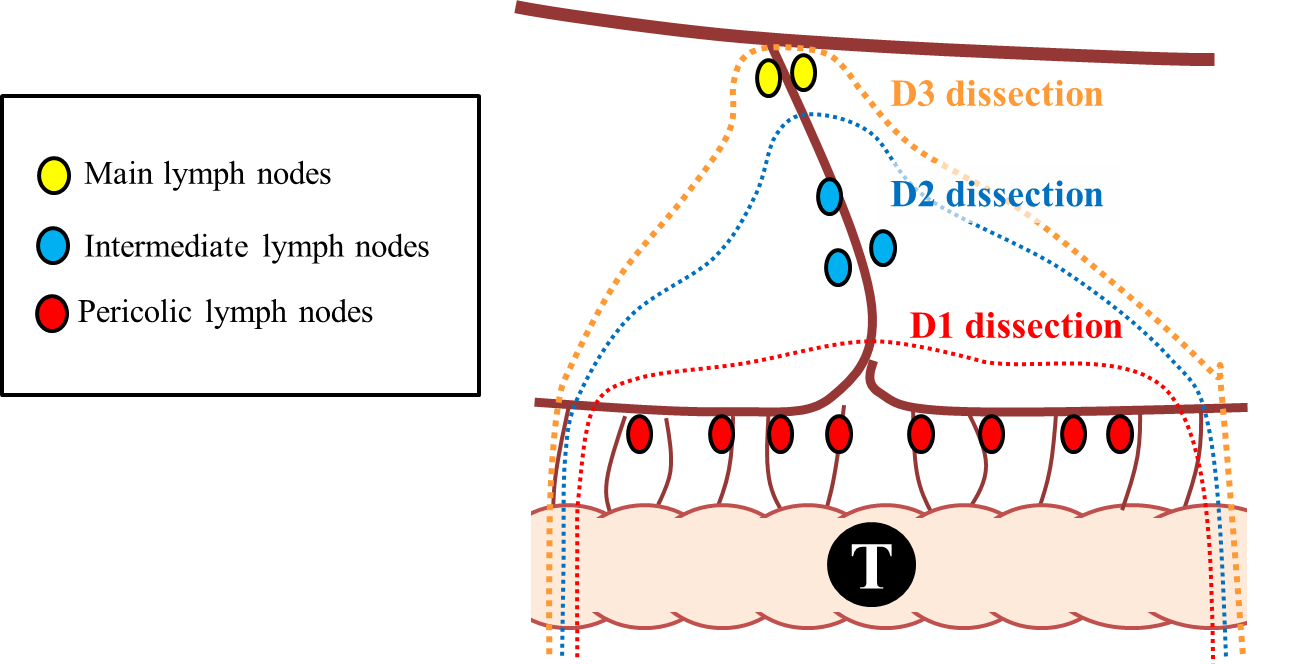
**

**Figure S2: CONSORT diagram**


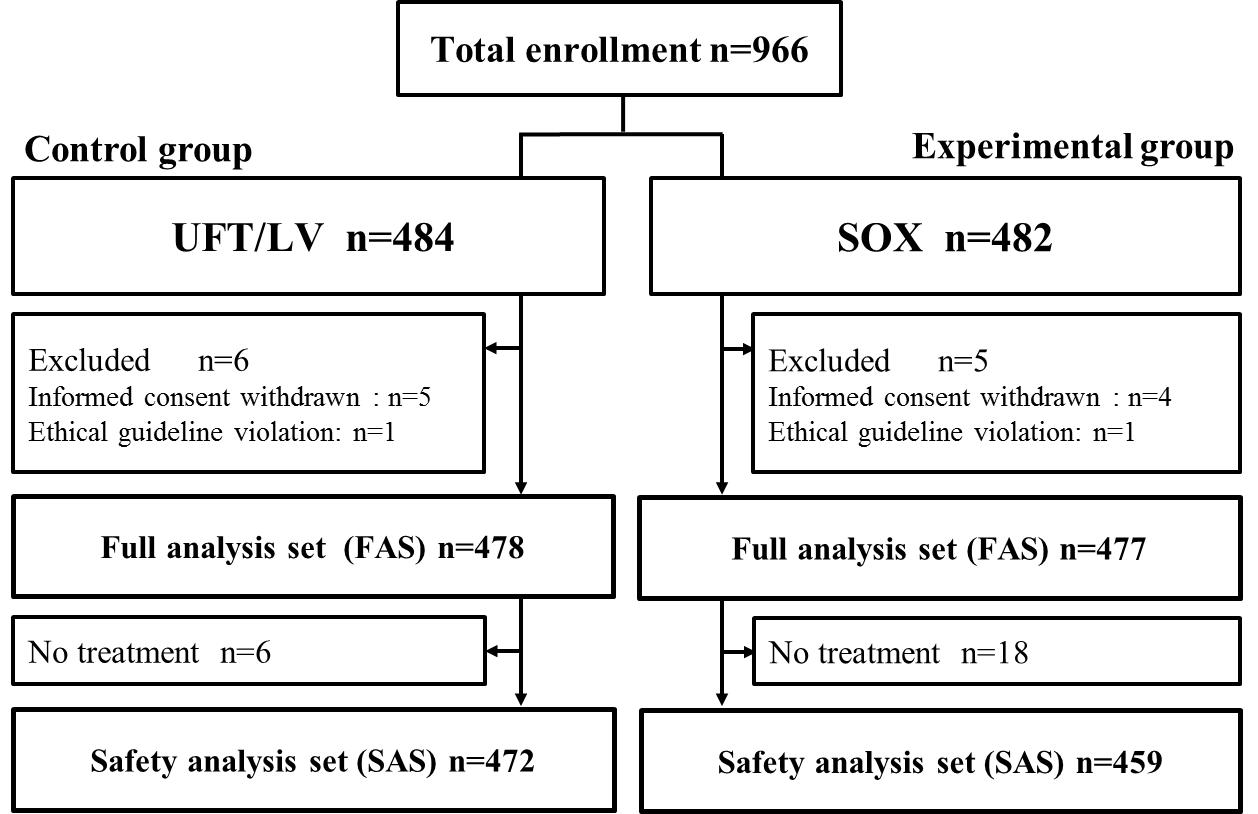


**Figure S3: Subgroup analyses of disease free survival**


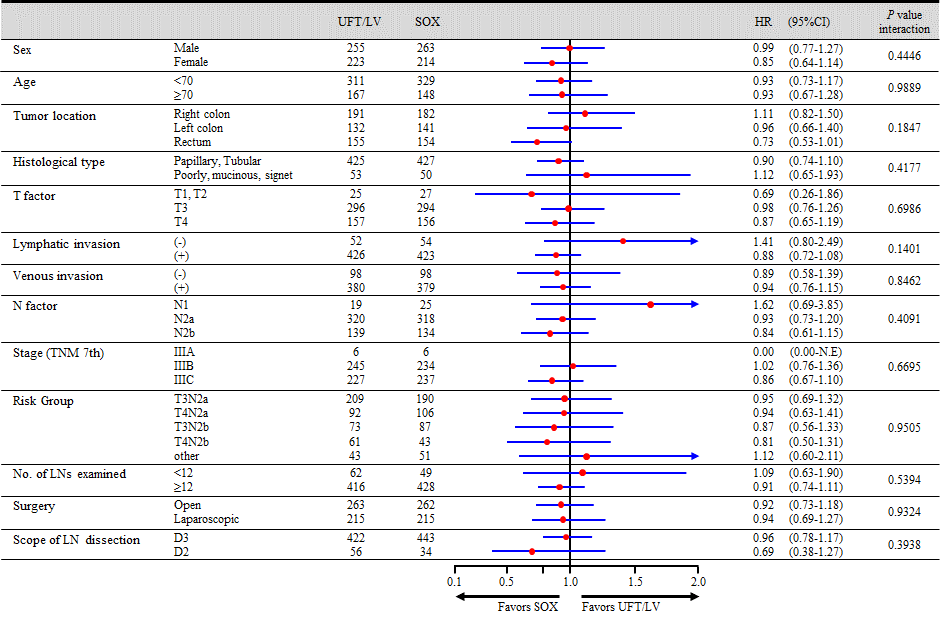

Supplement: Supplementary Figures [file mmc1.docx]
