# Supplementary material for: S-1 and oxaliplatin versus tegafur-uracil and leucovorin as post-operative adjuvant chemotherapy in patients with high-risk stage III colon cancer: updated 5-year survival of the phase III ACTS-CC 02 trial
Source: ESMO Open. 2021 Mar 11;6(2):100077. doi: 10.1016/j.esmoop.2021.100077 (PMC7966838; doi:10.1016/j.esmoop.2021.100077)
Supplement: Supplementary Appendix [file mmc2.docx]

**Supplementary Appendix**

**A list of participating institutions**

Japanese Red Cross Medical Center, National Kyushu Medical Center, Yokohama City University Medical Center, Hirosaki University Graduate School of Medicine, Gifu University Graduate School of Medicine, Hyogo College of Medicine, Ogaki Municipal Hospital, Iwate Prefectural Central Hospital, Gifu　Prefectural General Medical Center, Kurashiki Central Hospital, Sendai Kousei Hospital, Sendai Open Hospital, International Goodwill Hospital, Aizawa hospital, Hiroshima City Asa Citizens Hospital, South Miyagi Medical Center, Tokyo Medical and Dental University, Nihon University Itabashi Hospital, Nihon University Hospital, Mie Prefectural General Medical Center, Sasebo City General Hospital, Asahi General Hospital, Showa University Koto-Toyosu Hospital, Yokohama Medical Center, Hamamatsu University Hospital, Ageo Central General Hospital, Niigata City General Hospital, Japanese Red Cross Mito Hospital , Nippon Medical School Musashi Kosugi Hospital, Yokohama Municipal Citizen’s Hospital, Seirei Hamamatsu General Hospital, Japanese Red Cross Society Nagano Hospital, Gifu City Hospital , Kouseiren Takaoka Hospital, Fukui-ken Saiseikai Hospital, Matsusaka City Hospital, Steel Memorial Muroran Hospital, Nagaoka Chuo General Hospital, Toranomon Hospital , Showa University Hospital, Nippon Medical School Hospital, Saiseikai Yokohamashi Nanbu Hospital, Yokohama City University Hospital, Konan Kosei Hospital, Osaka Medical College Hospital, Otaru-Ekisaikai Hospital , Oji General Hospital , Kita-Fukushima Medical Center, Akita University, Kitasato University Medical Center, Omori Red Cross Hospital, Juntendo University Nerima Hospital, NTT Medical Center Tokyo, Higashiyamato Hospital, Kanagawa Cancer Center, Saiseikai Yokohamashi Tobu Hospital , Ibi Kosei Hospital , Nagano Municipal Hospital, Anjo Kosei Hospital, Toyota Kosei Hospital, Mie University Hospital, Japanese Red Cross Kyoto Daini Hospital , Kobe City Medical Center West Hospital, Matsue Redcross Hospital, Saiseikai Fukuoka General Hospital, Asahikawa Medical University, Sendai Medical Center, Osaki Citizen Hospital, Maebashi Red Cross Hospital, Niigata Prefecture Yoshida Hospital, Nagaoka Red Cross Hospital, Dokkyo Medical University Saitama Medical Center, Saitama Medical University, International Medical Center, Gunma Prefectural Cancer Center, Hitachi,Ltd.,Hitachi General Hospital, Tokyo Women's Medical University Hospital, Kawakita General Hospital, Musashino Red Cross Hospital, Tokyo Medical Center, Teikyo University Hospital, National Center for Global Health and Medicine, Fujisawa City Hospital, Fujinomiya City General Hospital, Matsunami General Hospital, Shiga University of Medical Science, Osaka City General Hpspital, Osaka International Cancer Institute, Kobe City Medical Center General Hospital, Miyoshi Central Hospital, Oita Red Cross Hospital, Coloproctology Center Takano Hospital, Sapporo City General Hospital, Kin-ikyo Chuo Hospital, Yamagata University Hospital, Gunma Saiseikai Maebashi Hospital, Dokkyo Medical University Hospital, Yuai Memorial Hospital, Tsukuba Medical Center Hospital, Itabashi Chuo Medical Center, Tokyo Metropolitan Cancer and Infectious Diseases Center, Komagome Hospital, St.Marianna University School of Medicine Hospital, Shizuoka General Hospital, Kanazawa Medical Center, Asama General Hospital, Gifu Prefectural Tajimi Hospital, Kizawa Memorial Hospital, Nagoya City University Hospital, Kyoto Okamoto Memorial Hospital, National Hospital Organization Kyoto Medical Center, Yamato Takada Municipal Hospital, Hashimoto Municipal Hospital, Sanda City Hospital, Kobe Rosai Hospital, Kawasaki Medical School Hospital, National Hospital Organization Kure Medical Center and Chugoku Cancer Center, Japan Community Health Care Organization Shimonoseki Medical Center, Tokushima University, Kochi Health Sciences Center, Kochi Medical School Hospital, Uwajima City Hospital, Social Insurance Tagawa Hospital, Saga-ken Medical Centre Koseikan, Oita Medical Center, Kurume University Hospital, Hamanomachi Hospital, Steel Memorial Yawata Hospital, Omuta City Hospital, Kumamoto Regional Medical Center, Kumamoto Rosai Hospital, Kagoshima City Hospital, Nakagami Hospital, Kobayashi Hospital, Yokote Municipal Hospital, Tohoku University Hospital, Sendai City Hospital, Shirakawa Kosei General Hospital, Sekishindo Hospital, Saiseikai Utsunomiya Hospital, Nishisaitama-chuo National Hospital, Teikyo University Chiba Medical Center, Tsuchiura Kyodo General Hospital, Juntendo Urayasu Hospital, Toho University Ohashi Medical Center, Ikegami General Hospital, Tokyo Medical University Hospital, Saiseikai Wakakusa Hospital, Sagamihara Hospital, Ashigarakami Hospital, Kanto Rosai Hospital, Kawasaki Municipal Tama Hospital, Saiseikai Takaoka Hospital, Toyama Red Cross Hospital, Komatsu Municipal Hospital, Hamamatsu Medical Center, Shimada Municipal Hospital, Shizuoka City Shizuoka Hospital, Fujita Health University, Japanese Red Cross Otsu Hospital, Nishijin Hospital, Kyoto Katsura Hospital, Suita Municipal Hospital, Osakafu Saiseikai Noe Hospital, Takatsuki General Hospital, Sakai City Medical Center, Hyogo　Prefectural　Awaji　Medical　Center, Hyogo Cancer Center, Kobe City Nishi-Kobe Medical Center, Shinko Hospital, Japanese Red Cross Society Himeji Hospital, Chugoku Central Hospoital, Tottori University Hospital, Yamaguchi University Hospital, Ehime University Hospital, Ehime Prefectural Central Hospital, Kagawa Prefectural Central Hospital, Kurume University Hospital, Nagasaki University Hospital, Nagasaki Medical Center, Miyazaki Prefectural Nobeoka Hospital, Kumamoto University Hospital, Japanese Red Cross Kumamoto Hospital, Asahikawa-Kosei General Hospital, Sapporo Medical University Hospital, Akita Red Cross Hospital, Akita City Hospital, Iwate Prefectural Chubu Hospital, Iwate Prefectural Isawa Hpspital, Miyagi Cancer Center, JCHO Sendai South Hospital, Tohoku Rosai Hospital, Fukushima Medical University Hospital, Fujioka General Hospital, National Hospital Organization Takasaki General Medical Center, Shuuwa General Hospital, Tsuchiura Kyodo General Hospital, New Tokyo Hospital, Chiba University Hospital, Chibaken Saiseikai Narashino Hospital, St. Luke's International Hospital, Tokyo-Kita Medical Center, Tokyo Metropolitan Tama Medical Center, Yokohama Sakae Kyosai Hospital, Yokohama City Minato Red Cross Hospital, Showa University Fujigaoka Hospital, Hiratsuka Kyosai Hospital, Sagamihara Kyodo Hospital, Kitasato University Hospital, Toyama Prefectural Central Hospital, Public Central Hospital of Matto Ishikawa, Keiju Medical Center, Japanese Red Cross Kanazawa Hospital, Ishikawa Prefectural Central Hospital, Kanazawa Medical University Hospital, Kaga Medical Center, Japanese Red Cross Fukui Hospital, Shinshu University Hospital, Yokoyama Memorial Hospital, Okazaki City Hospital, Meitetsu Hospital, Chubu Rosai Hospital, Kyoritsu General Hospital, Japanese Red Cross Nagoya Daini Hospital, Fujita Health University Bantane Hospital, JCHO Chukyo Hospital, Gifu University Hospital, Japanese Red Cross Ise Hospital, University Hospital Kyoto Prefectural University of Medicine, Osaka General Medical Center, Japanpost Osaka-kita Teishin Hospital, Toyonaka Municipal Hospital, Matsushita Memorial Hospital, Nippon Life Hospital, Kindai University Hospital, Saiseikai Suita Hospital, Kindai University Nara Hospital, Saiseikai Nara Hospital, Tenri Hospital, Japanese Red Cross Wakayama Medical Center, Rinku General Medical Center, Nara Medical University Hospital, Kansai Rosai Hospital, Kita-Harima Medical Center, Matsue City Hospital, Tsuyama Chuo Hospital, Okayama University Hospital, National Hospital Organization, Fukuyama Medical Center, Higashihiroshima　Medical Center, Tottori Prefectural Central Hospital, Tokushima Municipal Hospital, Anan Kyoei Hospital, Mitoyo General Hospital, Kochi National Hospital, Fukuoka University Hospital, Kyushu University Hospital, Kyushu University Hospital, Oita Prefectural Hospital, Karatsu Red Cross Hospital, Fukuoka University Chikushi Hospital, Kumamoto Medical Center, Kagoshima University Hospital
